# Cattle as Biological Indicators of *Echinococcus granulosus* Sensu Stricto in an Endemic Region from Chile

**DOI:** 10.3390/ani16121901

**Published:** 2026-06-19

**Authors:** Flery Fonseca-Salamanca, Angélica Melo, Juan Venegas, Marco Paredes, José Villanueva, Daniela Poo-Muñoz, Tamara Muñoz-Caro, Christian Herrera-George, Alejandro Hidalgo

**Affiliations:** 1 Molecular Immunoparasitology Laboratory, Center of Excellence in Medicina Translational Medicine (CEMT), Faculty of Medicine, Universidad de La Frontera, Temuco 4810296, Chile; flery.fonseca@ufrontera.cl (F.F.-S.); angelica.melo@ufrontera.cl (A.M.); jose_villanueva.rickemberg@hotmail.com (J.V.); 2 Department of Preclinical Science, Faculty of Medicine, Universidad de La Frontera, Temuco 4781176, Chile; christian.herrera@ufrontera.cl; 3 Molecular Phylogeny Laboratory, Cellular and Molecular Biology Program, Institute of Biomedical Science (ICBM), Faculty of Medicine, Universidad de Chile, Santiago 8380453, Chile; juanvenegas@uchile.cl; 4 Animal Biology Research Laboratory, Department of Basic Sciences, Faculty of Medicine, Universidad de La Frontera, Temuco 4811230, Chile; marco.paredes@ufrontera.cl; 5 Department of Agricultural Sciences and Natural Resources, Faculty of Agricultural and Environmental Sciences, Universidad de La Frontera, Temuco 4811230, Chile; daniela.poo@ufrontera.cl; 6 Faculty of Veterinary Medicine and Natural Resources, School of Veterinary Medicine, Universidad Santo Tomás, Talca 3460000, Chile; tmunoz6@santotomas.cl

**Keywords:** *Echinococcus granulosus*, cattle, hydatid cyst, *cox*1, Chile

## Abstract

Cystic echinococcosis (CE), caused by *Echinococcus granulosus* sensu lato (s.l.), is a significant zoonotic disease affecting livestock and public health worldwide, particularly in endemic regions such as La Araucanía, Chile. This study evaluated the role of cattle in the transmission dynamics of *E. granulosus* sensu stricto (s.s.) by morphologically and molecular characterizing hydatid cysts (HC) collected from cattle, sheep, pigs, and goats. A total of 123 cysts were obtained from a local slaughterhouse, with cattle contributing the majority of samples (*n* = 94). Fertility was highest in sheep (76.2%) and low in cattle (3.2%), while cysts from pigs and goats were infertile. PCR amplification and sequencing of the *cox*1 gene confirmed the predominance of genotype G1 (98.1%), with two additional haplotypes (Eg^B^ and Eg^C^) identified in cattle and sheep. Two cattle samples harbored genotype G3. Phylogenetic analyses grouped all sequences within the *E. granulosus* s.s. complex. The results corroborate the role of cattle as important sentinels for environmental surveillance of CE due to their exposure and traceability but highlight their lower competence in parasite transmission to definitive hosts compared with sheep. The genetic diversity observed aligns with previous findings in Chile, underscoring the epidemiological significance of *E. granulosus* s.s. and genotype G1 in the region.

## Simple Summary

Cystic echinococcosis is a parasitic disease that affects livestock and humans, and it remains a public health concern in southern Chile. It is caused by *Echinococcus granulosus*, a small tapeworm that inhabits the intestine of dogs and forms cysts in the organs of livestock and humans. This study investigated the role of cattle in the transmission of this parasite by analyzing hydatid cysts collected from cattle, sheep, pigs, and goats in the La Araucanía Region. Most cysts belonged to the common global strain known as genotype G1, while a few samples exhibited other related genetic types. Fertile cysts were mainly detected in sheep, whereas those from cattle were predominantly infertile, indicating that cattle do not effectively contribute to maintaining the parasite’s life cycle. Nevertheless, because cattle are frequently slaughtered and their origin can be easily traced, they serve as valuable sentinels for environmental surveillance of this disease. Understanding these infection patterns among livestock contributes to improving control strategies and reducing the risk of human infection in endemic areas.

## 1. Introduction

Cystic echinococcosis (CE), also known as hydatidosis, is a zoonotic disease caused by the larval stage of the cestode *Echinococcus granulosus* sensu lato (s.l.). It has a substantial impact on livestock production and public health in the regions where it occurs [1].

In Chile, CE is endemic, and in the La Araucanía Region, the disease exhibits particularly high incidence rates, along with the highest mortality rates in the country [2]. The sociodemographic characteristics of this area, with approximately 29.1% of its population living in rural settings (well above the national average), and an indigenous population of about 34.5% [3], contribute to maintaining traditional subsistence livestock farming systems that strongly influence the persistence of the parasite’s life cycle in this region. Additional contributing factors include the presence of dogs with limited or no veterinary care and the practice of home slaughter of livestock, all of which favor the persistence of the parasitosis [4,5].

Similar to other regions where CE occurs worldwide, in Chile *E. granulosus* sensu stricto (s.s.). is the predominant species. Within this complex, the G1 genotype (“sheep strain”) is by far the most prevalent in both animals and humans, highlighting its major epidemiological importance in maintaining the transmission cycle of the parasite in endemic areas. In contrast, genotype G3 (“buffalo strain”) has been reported less frequently [6,7,8,9]. Furthermore, *E. ortleppi* (G5), which exhibits higher fertility rates in cattle, has also been detected in Chile, although it occurs less frequently [8].

In livestock, both cattle and small ruminants, CE represents the second most frequent cause of viscera condemnation, primarily livers, after *Fasciola hepatica*. In cattle, it accounts for approximately 13–20% of the estimated annual confiscations, resulting in economic losses exceeding USD 1.3 million for this species alone [10,11].

Cattle are also an important species for CE surveillance. The main reasons include their environmental exposure and susceptibility to infection, their frequent slaughter throughout the country, and their zootechnical traceability, which facilitates access to samples and records of geographic origin [12,13,14]. Nevertheless, the susceptibility of cattle to *E. granulosus* s.s. infection does not correspond to their competence as intermediate hosts in the parasite’s life cycle. This is because the development of hydatid cysts in cattle exhibits low fertility compared to those in other hosts, such as sheep, particularly in areas where *E. granulosus* s.s. is the dominant complex [14,15,16]. This condition has been associated with the immune responses that different hosts develop against infection by circulating *E. granulosus* s.l. genotypes [17,18,19,20].

Based on this difference observed in cattle, the aim of this study was to evaluate the potential contribution of this species to the transmission dynamics of *E. granulosus* s.s. in an endemic area of Chile, through the morphological and molecular characterization of hydatid cysts in four livestock species.

## 2. Materials and Methods

### 2.1. Collection, Processing, and Morphological Characterization of Hydatid Cysts

From August to December of 2016, hydatid cysts were collected from four host species (cattle, sheep, pigs, and goats) at a slaughterhouse in the La Araucanía Region, Chile. For each animal, one cyst was donated by the facility, selecting the one with the largest relative size.

Samples were labeled with geographic origin, the organ from which the cyst was removed, and the sex of the animal, and were immediately transported to the laboratory in insulated containers. Fertility was determined by optical microscopy based on the presence or absence of protoscoleces or rostellar hooks in a drop of sediment obtained from the cyst content, examined at 100× total magnification. After recording these observations, samples were fixed in 70% ethanol, numbered, and stored at −20 °C until further processing.

### 2.2. DNA Extraction

Five hundred microliters of germinal membranes were extracted from each sample and individually transferred to 1.5 mL tubes. One milliliter of phosphate-buffered saline (PBS 1X, pH 7.4) was added, and the samples were centrifuged at 16,000× *g* at 4 °C for 5 min. This step was repeated three times. After each centrifugation, the supernatants were discarded, and the pellets were retained. Then, 600 µL of lysis buffer (10% SDS, 1 M Tris, and 0.5 M EDTA) and 15 µL of Proteinase K (Invitrogen^®^, Life Technologies, CA, USA. Concentration > 40 U/mg = 10 mg/mL) were added to the pellets, which were then incubated for 12 h at 58 °C [21].

Protein precipitation was performed by adding 400 µL of ammonium acetate at 4 °C to each tube containing the incubated mixture. The samples were gently mixed and incubated at 15 °C for 15 min. The samples were then centrifuged at 16,000× *g* for 15 min at 4 °C. Each supernatant was transferred to a new 1.5 mL tube containing 400 µL of chloroform, followed by centrifugation under the same conditions for 5 min.

The resulting supernatant was mixed with 600 µL of isopropanol and incubated for 12 h. After incubation, the tubes were centrifuged at 16,000× *g* at 4 °C for 10 min, and the supernatants were discarded. At this stage, the presence of a pellet was observed. Then, 600 µL of 70% ethanol was added, followed by a final centrifugation under the same previous conditions for 5 min, and the supernatant was discarded again.

Finally, the tubes were inverted to allow excess liquid to drain, and the DNA pellet was hydrated and stored indefinitely in TE buffer (10 mM Tris-HCl + 1 mM disodium EDTA; pH 8.0) at −20 °C until PCR assays were performed.

### 2.3. PCR Assays and Sequencing

PCR was performed in a final reaction volume of 40 µL, containing 20 µL of GoTaq^®^ Green Master Mix (Promega, Madison, WI, USA), 2 µL of forward primer (10 µM), 2 µL of reverse primer (10 µM), 2 µL of DNA template (>1 ng/µL), and nuclease-free water to complete the final volume.

The primers used were JB3 (5′-TTTTTTGGGCATCCTGAGGTTTAT-3′) and JB4.5 (5′-TAAAGAAAGAACATAATGAAAATG-3′), as described by Bowles et al. [22]. The PCR protocol consisted of an initial denaturation step at 94 °C for 1 min, followed by 40 cycles of 30 s at 94 °C (denaturation), 30 s at 51 °C (annealing), and 30 s at 70 °C (extension), with a final extension step at 72 °C for 10 min.

The presence and size of the amplified fragments were confirmed by electrophoresis on 2% agarose gels stained with GelRed, using 5 µL of PCR product from each sample.

Additionally, 30 µL of each reaction, along with the respective primers, were submitted to an external company for enzymatic purification and bidirectional sequencing (5′ to 3′ and 3′ to 5′) of the PCR products by capillary Sanger electrophoresis.

### 2.4. Phylogenetic Analysis

Sequence alignment and phylogenetic reconstruction were performed using the ClustalW algorithm implemented in BioEdit (https://bioedit.software.informer.com/7.2/ Accessed on 23 March 2025) and MEGA 6.0 software (https://www.megasoftware.net/ Accessed on 10 November 2024). Phylogenetic trees were constructed using the Neighbor-Joining (NJ) method with 1000 bootstrap replicates. Comparisons were made with *E. granulosus* s.l. genotype sequences available in GenBank.

Analyses were conducted using the Kimura 2-parameter model, assuming equal nucleotide frequencies, equal substitution rates, and a Gamma distribution to account for variable mutation rates among sites [23].

## 3. Results

### 3.1. Sample Collection

**Figure 1:**
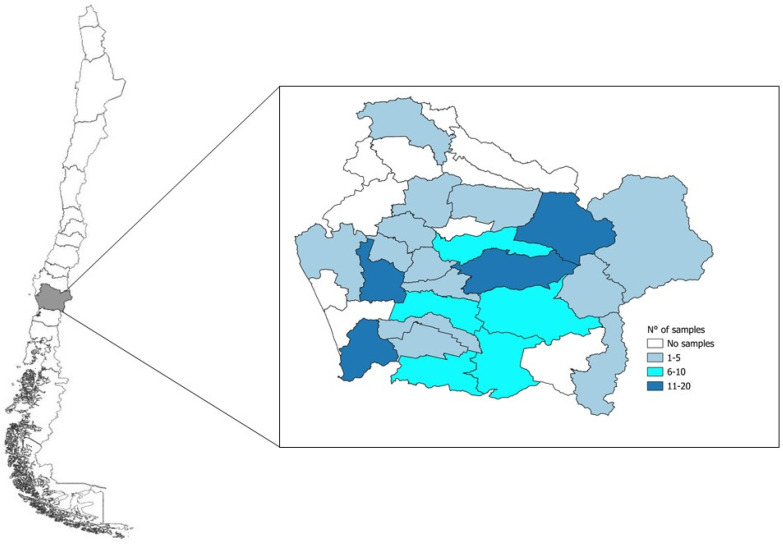
Map showing the distribution of hydatid cyst findings in four host animal species from various localities in the La Araucanía region, Chile.

A total of 121 hydatid cysts (HC) were collected from 121 animals belonging to four host species—cattle, sheep, goats, and pigs—originating from various locations in the La Araucanía region (Figure 1). The largest number of HC was obtained from cattle (*n* = 94), followed by sheep (*n* = 21), pigs (*n* = 4), and goats (*n* = 2).

Regarding the organs from which the samples were obtained, the majority of HC were collected from the lungs (*n* = 63), compared to hepatic cysts (*n* = 58), although the difference was not statistically significant. However, among the 21 cysts collected from sheep, 17 were found in the liver and only 4 in the lungs (Table 1).

**Figure 2:**
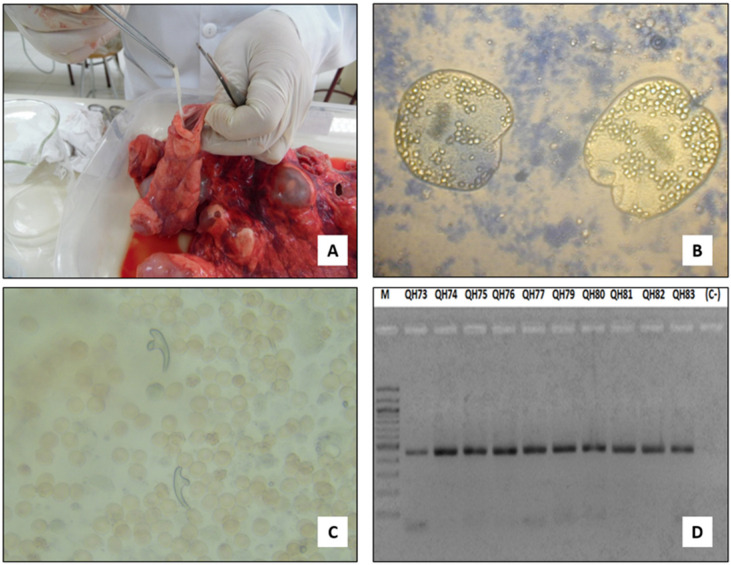
Representative images of selected aspects evaluated in hydatid cyst samples: (**A**) Extraction of germinal layer from a hydatid cyst. (**B**) Protoscoleces of *Echinococcus granulosus* extracted from a fertile hydatid cyst. (**C**) Rostellar hooks observed in the fluid from fertile cyst. (**D**) Demonstration of PCR amplification of a 445 bp fragment of the partial sequence of the *E. granulosus cox*1 gene in a set of hydatid cyst samples (QH73-QH83) on a 2% agarose gel. 100 bp molecular weight marker (M) and negative control with the reaction mixture used (C−).

Of the total 121 samples collected, 20 (16.5%) were fertile. The highest proportion of fertility per host species was observed in sheep, with 17 fertile cysts out of 21 collected (81.0%), in contrast to cattle, in which only 3 out of 94 (3.2%) were fertile. This condition was corroborated by the presence of protoscoleces and hooks in the material extracted from the hydatid cysts (Figure 2A–C). Viability was not assessed because most samples contained a low number of protoscoleces, precluding a reliable estimation. In pigs and goats, few infected animals were detected, and all HC were infertile.

### 3.2. PCR Assays for Cox1 Amplification

In 106 samples from the total, the expected fragment of approximately 445 bp was successfully amplified (Figure 2D). The 15 samples in which amplification failed were all derived from infertile cysts.

**Figure 3:**
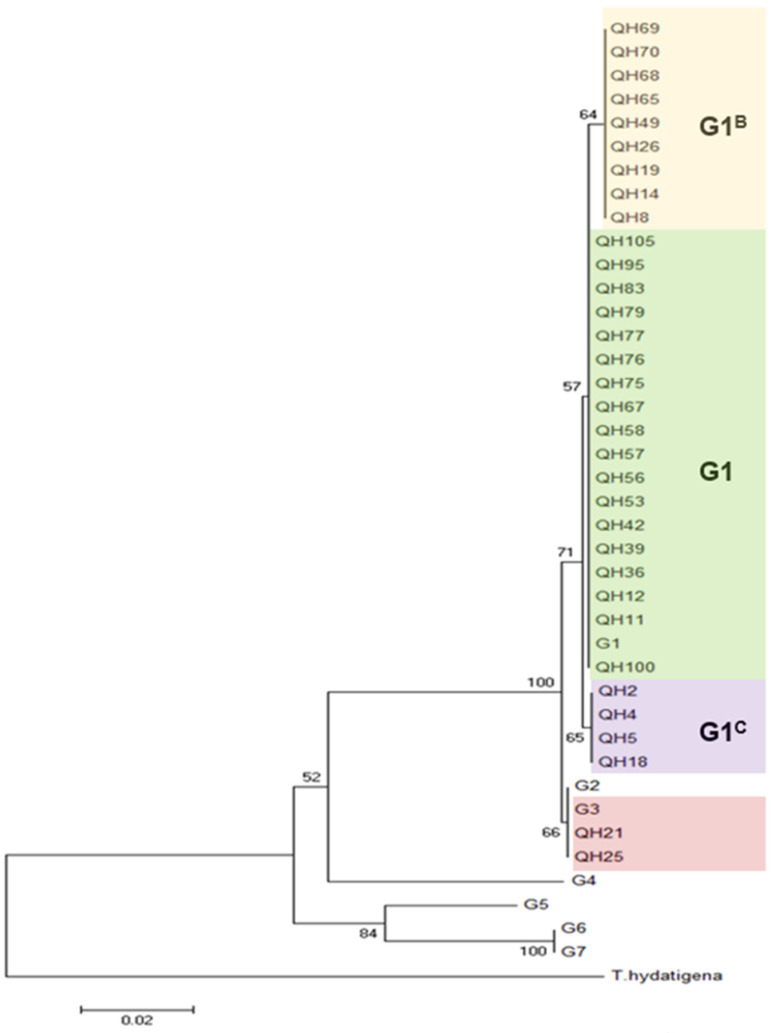
Phylogenetic analysis of the diversity of *Echinococcus granulosus* sensu stricto in hydatid cyst samples collected in La Araucanía region of Chile. Reference genotype sequences: G1 (AB271235.1), G3 (DQ856466.1)/G2 (KJ162562.1), G4 (EF143834.1), G5 (KC415063.1), G6 (KF731906.1), G7 (KJ556997.1) and *Taenia hydatigena* as the outgroup. Haplotypes shown in the figure: Eg^B^ (G1 ^B^) and Eg^C^ (G1^C^).

Analysis of these sequences revealed that 104 of the amplified samples (98.1%) corresponded to genotype G1. Among these, 75 were identified as haplotype 1, recognized as the reference sequence for G1. However, in 29 (24.3%) of them, mutations were detected at specific polymorphic sites, suggesting two additional haplotypes within genotype G1 (Figure 3, Supplementary File S1). In 23 of these samples—19 samples from cattle and 4 from sheep—a thymine-to-cytosine substitution (T→C) was detected at position 75, designating this haplotype as Eg^B^ (Figure 3).

In the remaining 6 samples (5.6%)—3 from cattle and 3 from sheep—a guanine-to-adenine substitution (G→A) was identified at position 246, which designated this haplotype as Eg^C^ (Figure 3).

Isolates QH21 and QH25, corresponding to two cattle isolates from the indigenous locality of Nueva Imperial, exhibited polymorphisms consistent with genotype G3, occurring at two sites: a cytosine-to-thymine substitution (C→T) at position 66 and a thymine-to-cytosine substitution (T→C) at position 257.

Overall, 100% of the sequences corresponded to the *E. granulosus* s.s. species complex, with no other species or genotypes of *E. granulosus* s.l. identified.

As confirmation, the phylogenetic analysis revealed the formation of a common node clustering *E. granulosus* s.s. (Genotypes G1, G2, and G3) with 100% statistical support (Figure 3). In turn, the cluster corresponding to genotype G1 displayed three subnodes separating the three possible haplotypes identified, consistent with the detected mutations. Additionally, samples QH21 and QH25 were grouped with the reference sequence of genotype G3.

## 4. Discussion

In Chile, studies on cystic echinococcosis (CE) in livestock have been conducted most frequently in cattle, consistent with the fact that most national slaughterhouses are equipped for processing this species [6,7,8]. This finding aligns with our study, in which sheep, pigs, and goats were sampled in decreasing and significantly lower numbers. This pattern is consistent with national records of slaughter volumes and condemnations reported by the Agricultural and Livestock Service (SAG) in Chile. In the La Araucanía region in particular, according to the SAG report for the sampling year, the prevalence of cystic echinococcosis was 85.3% in cattle, 13.8% in sheep, and 0.3% in goats [11].

Furthermore, unlike cattle, the slaughter age of sheep, goats, and pigs is relatively young (<6 months), a period generally insufficient for the development of hydatid cysts (HC), considering that the formation of a vesicle measuring between 50 and 100 mm in diameter can take 4 to 6 months from the time of infection, depending on the parasite species involved [24].

The fertility observed in the hydatid cysts collected from cattle in this study was even lower than the results reported in Chile by Muñoz and Sievers [25], who estimated a fertility rate of 26.1%. Similarly, studies conducted in other livestock regions worldwide, such as Ethiopia with 23.9% [26] and 38.8% [27], and various locations in Iran with 5.3% [15], 14.85% [28], and 19.0% [29], showed fertility rates higher than those found in our study. In contrast, other investigations reported similar or lower percentages, such as 2.5% in Iran [30] and 0% in Libya [31]. In all these studies, the fertility rates observed in cattle were consistently lower than those found in sheep cysts.

According to Rinaldi et al. [32], the low fertility of hydatid cysts produced by *E. granulosus* s.s. in cattle makes them inefficient in the transmission of *E. granulosus* s.s. However, due to their susceptibility and the fact that most cattle graze directly on pastures, they serve as valuable indicators for assessing the presence of this parasite in the environment.

In contrast, the fertility of hydatid cysts in sheep has been reported to be significantly higher than in other intermediate hosts, making sheep the most epidemiological important species in the transmission of *E. granulosus* s.s. to definitive canine host, particularly domestic dogs. This is inferred from the generally high fertility of cysts, which typically ranges from 40 to 80% [15,28,29,30].

Despite the low fertility of hydatid cysts produced by *E. granulosus* s.s. in cattle, which reduces their direct efficacy in transmitting the parasite, cattle remain integral to the cycle as sentinel hosts. This role is largely attributed to their grazing habits, which facilitate the detection and monitoring of the parasite’s presence within an ecosystem [32].

Furthermore, the widespread distribution of cattle across diverse agricultural landscapes amplifies their value as indicators of *E. granulosus* s.s. presence. The extensive grazing ranges of cattle (a common practice in southern Chile) increase the likelihood of encountering parasite eggs, thereby providing a comprehensive reflection of the parasite’s geographical distribution and prevalence. This characteristic is particularly advantageous for epidemiological surveillance, enabling researchers to assess and map the parasite’s presence across different regions and, in turn, inform public health strategies and interventions.

In contrast, no fertility was observed in the cysts collected from pigs and goats. Additionally, the low number of samples collected from these species did not allow for a conclusive analysis of this condition, although previous studies have reported low fertility in these species compared with sheep and cattle in geographic areas where *E. granulosus* s.s. also predominates [16,29,33,34,35].

Several authors suggest that the apparent inability of *Echinococcus* spp. to generate viable infections in certain host species that develop infertile hydatid cysts may represent an attempt at adaptation and co-evolution over time rather than a complete failure [36,37].

The results obtained through the cox1 barcode analysis showed that *E. granulosus* s.s. was the only species-complex identified, with genotype G1 being the most frequent. These findings are consistent with previous studies conducted in Chile [6,7,8].

The prevalence of genotype G1 among human cases of echinococcosis indicates its higher zoonotic potential compared to other genotypes, such as G3. This heightened transmissibility is likely due to several factors, including its adaptability to a wide range of intermediate hosts, which facilitates its propagation across diverse ecological settings. The adaptability of genotype G1 enhances its capacity to complete its life cycle, thereby increasing the likelihood of human infection, particularly in regions where livestock management practices intersect with human habitation [32].

Within genotype G1, three different haplotypes were identified in this study. Although no official nomenclature exists for these variants, the most frequent was the so-called haplotype 1 (Eg01), which corresponds to the holotype sequence originally described by Bowles et al. [22] and is also the most common worldwide [13,38,39,40].

The second most frequent haplotype was provisionally named Eg^B^. This variant has been previously described in hydatid cysts from Peru [41], Algeria [42] and isolates from the Middle East [43]. In Chile, this mutation has also been reported [7,8], which could represent potential evidence of the historical and geographical displacement of different lineages within *E. granulosus* s.s., associated with the origin, development, and expansion of livestock farming, considering its presence in the Old World.

Additionally, the isolates designated as Eg^C^ exhibited a mutation not previously described in *E. granulosus* s.s. but located at the same site in the *cox*1 sequence of *E. ortleppi* (G5) and *E. canadensis* (G6 and G7), although with a different nucleotide (Thymine), suggesting that this site may be prone to mutations.

Furthermore, the finding of two cattle with infertile pulmonary cysts matching genotype G3, one of the genotypes within the *E. granulosus* s.s. complex, and the presence of this genotype, first described in Chile by Espinoza et al. [6] in pulmonary cysts from two cattle originating from the same geographic region, suggests that this genotype, although of low prevalence, continues to circulate in this area.

Corrêa et al. [8] identified the presence of genotype G5 (*E. ortleppi*), genotype G3, and 11 different haplotypes in cattle. Although the geographic origin of these samples was not specified (probably because the collection took place at a centralized slaughterhouse receiving animals from across the country). Similarly, Álvarez-Rojas et al. [7] reported high haplotype microdiversity within *E. granulosus* s.s. from samples originating in various regions of Chile, based on a longer *cox*1 gene sequence (1600 bp).

Molecular analyses have determined that *E. granulosus* s.s. with genotype G1 is the most frequent in humans and animals in South America [36,40,41,44]. However, in other South American countries, in addition to *E. granulosus* s.s., other genotypes associated with cattle have been reported, such as *E. ortleppi* and *E. canadensis* in southern Brazil [36,45], and *E. ortleppi* in Bolivia, where all cysts associated with this genotype were fertile [46]. Furthermore, *E. ortleppi* and *E. canadensis* have also been identified in cysts obtained from humans in Argentina [47]. This is an aspect that should be further explored in Chile, as the lower genotype diversity observed compared to other South American regions may be related to Chile’s relative geographic isolation, defined by natural barriers such as the Atacama Desert to the north, the Andes Mountains to the east, the Pacific Ocean to the west, and the ice fields of the far south.

In Chile, molecular studies conducted to date have shown that *E. granulosus* s.s. is the taxonomic group with the highest prevalence, with genotype G1 being the most frequent within this complex [6,7,8,9]. Other genotypes, such as *E. ortleppi* (G5), have also been described, but with low prevalence [7].

Dog presence, whether free-roaming or shepherd dogs, has been globally associated with the maintenance of the *E. granulosus* transmission cycle [48,49]. In Chile, in particular, the dog population has reached considerable numbers; more than 8 million owned dogs in the country [50], with the majority being free-roaming in peri-urban and rural areas. This situation poses a major challenge for the control of parasitic cycles such as that of *E. granulosus*. Therefore, population management and sanitary control measures are essential to reduce the risk of transmission to livestock and humans [49,51].

## 5. Conclusions

The *E. granulosus* s.s. complex was the only member of *E. granulosus* s.l. detected among the four sampled host species in the La Araucanía region, with genotype G1 being the most frequent and genotype G3 the least frequent. Cattle contributed the highest number of HC, but their fertility was comparatively low relative to that observed in sheep. These findings suggest that, while cattle serve as valuable sentinel hosts for environmental surveillance of cystic echinococcosis (CE), they play a limited role in the transmission cycle of *E. granulosus*.

## Figures and Tables

**Table 1:** Distribution and fertility in hydatid cyst samples collected from 121 livestock animals of different species in La Araucanía region, Chile.

Host	N° Samples	Fertile Cyst *n* (%)	Location (*n*)		Fertile Cyst *n* (%)
Cattle	94	3 (3.2)	Liver	39	3 (100)
			Lung	55	0 (0.0)
Sheep	21	17 (81.0)	Liver	17	13 (76.5)
			Lung	4	4 (23.5)
Pigs	4	0 (0.0)	Liver	2	0 (0.0)
			Lung	2	0 (0.0)
Goats	2	0 (0.0)	Liver	0	0 (0.0)
			Lung	2	0 (0.0)

## Data Availability

The raw data supporting the conclusions of this article will be made available by the authors on request. (Author Alejandro Hidalgo (alejandro.hidalgo@ufrontera.cl) can be contacted for data availability.)

## Supplementary Materials

The following supporting information can be downloaded at: https://www.mdpi.com/article/10.3390/ani16121901/s1, Table S1: Polymorphisms in a partial sequence from the *Echinococcus granulosus* s.l. cox1 gene. The numbers represent the polymorphic sites within the barcode sequence proposed for the diversity analysis of *E. granulosus* s.l. genotypes [21]. Matches for each nucleotide in the analysed samples (QH01 to QH121) are represented by a dot. The first seven are the genotypes reference sequences correspond to the following GenBank accession numbers: G1 (AB271235.1), G2* (KJ162562.1), G3 (DQ856466.1), G4 (EF143834.1), G5 (KC415063.1), G6 (KF731906.1) and G7 (KJ556997.1). * It is currently considered a microvariant of the G3 genotype; Table S2: Samples of hydatid cysts collected from various sources in the Araucanía region. Details include the name of the sample, the animal species from which it was collected, the organ, fertility, viability, whether *cox*1 markers were amplified, and the genotype or haplotype.
