# Supplementary material for: Cattle as Biological Indicators of Echinococcus granulosus Sensu Stricto in an Endemic Region from Chile
Source: Animals (Basel). 2026 Jun 19;16(12):1901. doi: 10.3390/ani16121901 (PMC13296003; doi:10.3390/ani16121901)
Supplement: Supplementary file 1 [file animals-16-01901-s001.zip › Supplementary File S1.pdf]

**Supplementary Table S1**: Polymorphisms in a partial sequence from the *Echinococcus granulosus* s.l. *cox*1 gene. The numbers represent the polymorphic sites within the barcode sequence proposed for the diversity analysis of *E. granulosus* s.l. genotypes [21]. Matches for each nucleotide in the analysed samples (QH01 to QH121) are represented by a dot. The first seven are the genotypes reference sequences correspond to the following GenBank accession numbers: G1 (AB271235.1), G2* (KJ162562.1), G3 (DQ856466.1), G4 (EF143834.1), G5 (KC415063.1), G6 (KF731906.1) and G7 (KJ556997.1). * It is currently considered a microvariant of the G3 genotype

| | 56 | 57 | 66 | 78 | 105 | 108 | 111 | 114 | 123 | 144 | 147 | 153 | 159 | 174 | 195 | 216 | 228 | 237 | 240 | 246 | 248 | 256 | 257 |
|---|---|---|---|---|---|---|---|---|---|---|---|---|---|---|---|---|---|---|---|---|---|---|---|
| G1 | C | G | C | G | G | T | C | G | T | G | G | G | G | C | G | G | A | G | G | G | G | G | T |
| G2 | T | · | T | · | · | · | · | · | · | · | · | · | · | · | · | · | · | · | · | · | · | · | C |
| G3 | · | · | T | · | · | · | · | · | · | · | · | · | · | · | · | · | · | · | · | · | · | · | C |
| G4 | · | · | T | · | A | A | T | · | G | · | · | · | · | T | A | T | G | · | A | · | A | A | A |
| G5 | · | · | T | A | A | · | T | T | · | · | · | T | · | T | T | · | G | A | · | T | A | C | G |
| G6 | · | · | T | · | · | · | T | T | A | A | A | · | T | T | T | · | G | A | · | T | A | · | C |
| G7 | · | · | T | · | · | · | T | T | A | A | A | · | T | T | T | · | G | A | · | T | A | · | C |
| QH1 | · | · | · | · | · | · | · | · | · | · | · | · | · | · | · | · | · | · | · | · | · | · | · |
| QH2 | · | · | · | · | · | · | · | · | · | · | · | · | · | · | · | · | · | · | · | A | · | · | · |
| QH4 | · | · | · | · | · | · | · | · | · | · | · | · | · | · | · | · | · | · | · | A | · | · | · |
| QH5 | · | · | · | · | · | · | · | · | · | · | · | · | · | · | · | · | · | · | · | A | · | · | · |
| QH7 | · | · | · | · | · | · | · | · | · | · | · | · | · | · | · | · | · | · | · | · | · | · | · |
| QH8 | · | · | · | C | · | · | · | · | · | · | · | · | · | · | · | · | · | · | · | · | · | · | · |
| QH10 | · | · | · | · | · | · | · | · | · | · | · | · | · | · | · | · | · | · | · | · | · | · | · |
| QH11 | · | · | · | · | · | · | · | · | · | · | · | · | · | · | · | · | · | · | · | · | · | · | · |
| QH12 | · | · | · | · | · | · | · | · | · | · | · | · | · | · | · | · | · | · | · | · | · | · | · |
| QH13 | · | · | · | · | · | · | · | · | · | · | · | · | · | · | · | · | · | · | · | · | · | · | · |
| QH14 | · | · | · | C | · | · | · | · | · | · | · | · | · | · | · | · | · | · | · | · | · | · | · |
| QH15 | · | · | · | · | · | · | · | · | · | · | · | · | · | · | · | · | · | · | · | · | · | · | · |
| QH16 | · | · | · | · | · | · | · | · | · | · | · | · | · | · | · | · | · | · | · | · | · | · | · |
| QH17 | · | · | · | · | · | · | · | · | · | · | · | · | · | · | · | · | · | · | · | · | · | · | · |
| QH18 | · | · | · | · | · | · | · | · | · | · | · | · | · | · | · | · | · | · | · | A | · | · | · |
| QH19 | · | · | · | C | · | · | · | · | · | · | · | · | · | · | · | · | · | · | · | · | · | · | · |
| QH20 | · | · | · | · | · | · | · | · | · | · | · | · | · | · | · | · | · | · | · | · | · | · | · |
| QH21 | · | · | T | · | · | · | · | · | · | · | · | · | · | · | · | · | · | · | · | · | · | · | C |
| QH22 | · | · | · | · | · | · | · | · | · | · | · | · | · | · | · | · | · | · | · | · | · | · | · |
| QH23 | · | · | · | · | · | · | · | · | · | · | · | · | · | · | · | · | · | · | · | · | · | · | · |
| QH24 | · | · | · | · | · | · | · | · | · | · | · | · | · | · | · | · | · | · | · | · | · | · | · |
| QH25 | · | · | T | · | · | · | · | · | · | · | · | · | · | · | · | · | · | · | · | · | · | · | C |
| QH26 | · | · | · | C | · | · | · | · | · | · | · | · | · | · | · | · | · | · | · | · | · | · | · |
| QH27 | · | · | · | C | · | · | · | · | · | · | · | · | · | · | · | · | · | · | · | · | · | · | · |
| QH28 | · | · | · | · | · | · | · | · | · | · | · | · | · | · | · | · | · | · | · | · | · | · | · |
| QH29 | · | · | · | · | · | · | · | · | · | · | · | · | · | · | · | · | · | · | · | · | · | · | · |
| QH31 | · | · | · | · | · | · | · | · | · | · | · | · | · | · | · | · | · | · | · | · | · | · | · |
| QH32 | · | · | · | · | · | · | · | · | · | · | · | · | · | · | · | · | · | · | · | · | · | · | · |
| QH33 | · | · | · | · | · | · | · | · | · | · | · | · | · | · | · | · | · | · | · | · | · | · | · |
| QH34 | · | · | · | · | · | · | · | · | · | · | · | · | · | · | · | · | · | · | · | · | · | · | · |

| | | | | | | | | | | | | | | | | | | | | | | | |
|---|---|---|---|---|---|---|---|---|---|---|---|---|---|---|---|---|---|---|---|---|---|---|---|
| QH35 | . | . | . | . | . | . | . | . | . | . | . | . | . | . | . | . | . | . | . | . | . | . | . |
| QH36 | . | . | . | . | . | . | . | . | . | . | . | . | . | . | . | . | . | . | . | . | . | . | . |
| QH37 | . | . | . | C | . | . | . | . | . | . | . | . | . | . | . | . | . | . | . | . | . | . | . |
| QH38 | . | . | . | . | . | . | . | . | . | . | . | . | . | . | . | . | . | . | . | . | . | . | . |
| QH39 | . | . | . | . | . | . | . | . | . | . | . | . | . | . | . | . | . | . | . | . | . | . | . |
| QH40 | . | . | . | . | . | . | . | . | . | . | . | . | . | . | . | . | . | . | . | A | . | . | . |
| QH41 | . | . | . | . | . | . | . | . | . | . | . | . | . | . | . | . | . | . | . | . | . | . | . |
| QH42 | . | . | . | . | . | . | . | . | . | . | . | . | . | . | . | . | . | . | . | . | . | . | . |
| QH43 | . | . | . | . | . | . | . | . | . | . | . | . | . | . | . | . | . | . | . | . | . | . | . |
| QH44 | . | . | . | . | . | . | . | . | . | . | . | . | . | . | . | . | . | . | . | . | . | . | . |
| QH47 | . | . | . | . | . | . | . | . | . | . | . | . | . | . | . | . | . | . | . | A | . | . | . |
| QH49 | . | . | . | C | . | . | . | . | . | . | . | . | . | . | . | . | . | . | . | . | . | . | . |
| QH50 | . | . | . | C | . | . | . | . | . | . | . | . | . | . | . | . | . | . | . | . | . | . | . |
| QH51 | . | . | . | . | . | . | . | . | . | . | . | . | . | . | . | . | . | . | . | . | . | . | . |
| QH53 | . | . | . | . | . | . | . | . | . | . | . | . | . | . | . | . | . | . | . | . | . | . | . |
| QH55 | . | . | . | . | . | . | . | . | . | . | . | . | . | . | . | . | . | . | . | . | . | . | . |
| QH56 | . | . | . | . | . | . | . | . | . | . | . | . | . | . | . | . | . | . | . | . | . | . | . |
| QH57 | . | . | . | . | . | . | . | . | . | . | . | . | . | . | . | . | . | . | . | . | . | . | . |
| QH58 | . | . | . | . | . | . | . | . | . | . | . | . | . | . | . | . | . | . | . | . | . | . | . |
| QH59 | . | . | . | . | . | . | . | . | . | . | . | . | . | . | . | . | . | . | . | . | . | . | . |
| QH61 | . | . | . | . | . | . | . | . | . | . | . | . | . | . | . | . | . | . | . | . | . | . | . |
| QH64 | . | . | . | C | . | . | . | . | . | . | . | . | . | . | . | . | . | . | . | . | . | . | . |
| QH65 | . | . | . | C | . | . | . | . | . | . | . | . | . | . | . | . | . | . | . | . | . | . | . |
| QH66 | . | . | . | C | . | . | . | . | . | . | . | . | . | . | . | . | . | . | . | . | . | . | . |
| QH67 | . | . | . | . | . | . | . | . | . | . | . | . | . | . | . | . | . | . | . | . | . | . | . |
| QH68 | . | . | . | . | . | . | . | . | . | . | . | . | . | . | . | . | . | . | . | . | . | . | . |
| QH69 | . | . | . | C | . | . | . | . | . | . | . | . | . | . | . | . | . | . | . | . | . | . | . |
| QH70 | . | . | . | C | . | . | . | . | . | . | . | . | . | . | . | . | . | . | . | . | . | . | . |
| QH71 | . | . | . | C | . | . | . | . | . | . | . | . | . | . | . | . | . | . | . | . | . | . | . |
| QH73 | . | . | . | . | . | . | . | . | . | . | . | . | . | . | . | . | . | . | . | . | . | . | . |
| QH74 | . | . | . | . | . | . | . | . | . | . | . | . | . | . | . | . | . | . | . | . | . | . | . |
| QH75 | . | . | . | . | . | . | . | . | . | . | . | . | . | . | . | . | . | . | . | . | . | . | . |
| QH76 | . | . | . | . | . | . | . | . | . | . | . | . | . | . | . | . | . | . | . | . | . | . | . |
| QH77 | . | . | . | . | . | . | . | . | . | . | . | . | . | . | . | . | . | . | . | . | . | . | . |
| QH79 | . | . | . | . | . | . | . | . | . | . | . | . | . | . | . | . | . | . | . | . | . | . | . |
| QH80 | . | . | . | . | . | . | . | . | . | . | . | . | . | . | . | . | . | . | . | . | . | . | . |
| QH81 | . | . | . | . | . | . | . | . | . | . | . | . | . | . | . | . | . | . | . | . | . | . | . |
| QH82 | . | . | . | . | . | . | . | . | . | . | . | . | . | . | . | . | . | . | . | . | . | . | . |
| QH83 | . | . | . | . | . | . | . | . | . | . | . | . | . | . | . | . | . | . | . | . | . | . | . |
| QH84 | . | . | . | . | . | . | . | . | . | . | . | . | . | . | . | . | . | . | . | . | . | . | . |
| QH86 | . | . | . | C | . | | . | . | . | . | . | . | . | . | . | . | . | . | . | . | . | . | . |
| QH87 | . | . | . | . | . | . | . | . | . | . | . | . | . | . | . | . | . | . | . | . | . | . | . |
| QH88 | . | . | . | C | . | . | . | . | . | . | . | . | . | . | . | . | . | . | . | . | . | . | . |
| QH89 | . | . | . | C | . | . | . | . | . | . | . | . | . | . | . | . | . | . | . | . | . | . | . |
| QH90 | . | . | . | . | . | . | . | . | . | . | . | . | . | . | . | . | . | . | . | . | . | . | . |
| QH91 | . | . | . | . | . | . | . | . | . | . | . | . | . | . | . | . | . | . | . | . | . | . | . |
| QH92 | . | . | . | . | . | . | . | . | . | . | . | . | . | . | . | . | . | . | . | . | . | . | . |
| QH93 | . | . | . | . | . | . | . | . | . | . | . | . | . | . | . | . | . | . | . | . | . | . | . |

| | | | | | | | | | | | | | | | | | | | | | | | |
|---|---|---|---|---|---|---|---|---|---|---|---|---|---|---|---|---|---|---|---|---|---|---|---|
| QH94 | . | . | . | C | . | . | . | . | . | . | . | . | . | . | . | . | . | . | . | . | . | . | . |
| QH95 | . | . | . | . | . | . | . | . | . | . | . | . | . | . | . | . | . | . | . | . | . | . | . |
| QH96 | . | . | . | . | . | . | . | . | . | . | . | . | . | . | . | . | . | . | . | . | . | . | . |
| QH97 | . | . | . | . | . | . | . | . | . | . | . | . | . | . | . | . | . | . | . | . | . | . | . |
| QH98 | . | . | . | C | . | . | . | . | . | . | . | . | . | . | . | . | . | . | . | . | . | . | . |
| QH99 | . | . | . | . | . | . | . | . | . | . | . | . | . | . | . | . | . | . | . | . | . | . | . |
| QH100 | . | . | . | . | . | . | . | . | . | . | . | . | . | . | . | . | . | . | . | . | . | . | . |
| QH101 | . | . | . | . | . | . | . | . | . | . | . | . | . | . | . | . | . | . | . | . | . | . | . |
| QH102 | . | . | . | . | . | . | . | . | . | . | . | . | . | . | . | . | . | . | . | . | . | . | . |
| QH103 | . | . | . | . | . | . | . | . | . | . | . | . | . | . | . | . | . | . | . | . | . | . | . |
| QH104 | . | . | . | . | . | . | . | . | . | . | . | . | . | . | . | . | . | . | . | . | . | . | . |
| QH105 | . | . | . | . | . | . | . | . | . | . | . | . | . | . | . | . | . | . | . | . | . | . | . |
| QH106 | . | . | . | . | . | . | . | . | . | . | . | . | . | . | . | . | . | . | . | . | . | . | . |
| QH107 | . | . | . | . | . | . | . | . | . | . | . | . | . | . | . | . | . | . | . | . | . | . | . |
| QH108 | . | . | . | **C** | . | . | . | . | . | . | . | . | . | . | . | . | . | . | . | . | . | . | . |
| QH109 | . | . | . | . | . | . | . | . | . | . | . | . | . | . | . | . | . | . | . | . | . | . | . |
| QH110 | . | . | . | . | . | . | . | . | . | . | . | . | . | . | . | . | . | . | . | . | . | . | . |
| QH111 | . | . | . | . | . | . | . | . | . | . | . | . | . | . | . | . | . | . | . | . | . | . | . |
| QH112 | . | . | . | C | . | | . | . | . | . | . | . | . | . | . | . | . | . | . | . | . | . | . |
| QH113 | . | . | . | . | . | . | . | . | . | . | . | . | . | . | . | . | . | . | . | . | . | . | . |
| QH114 | . | . | . | . | . | . | . | . | . | . | . | . | . | . | . | . | . | . | . | . | . | . | . |
| QH115 | . | . | . | . | . | . | . | . | . | . | . | . | . | . | . | . | . | . | . | . | . | . | . |
| QH116 | . | . | . | . | . | . | . | . | . | . | . | . | . | . | . | . | . | . | . | . | . | . | . |
| QH117 | . | . | . | **C** | . | . | . | . | . | . | . | . | . | . | . | . | . | . | . | . | . | . | . |
| QH118 | . | . | . | . | . | . | . | . | . | . | . | . | . | . | . | . | . | . | . | . | . | . | . |
| QH119 | . | . | . | . | . | . | . | . | . | . | . | . | . | . | . | . | . | . | . | . | . | . | . |
| QH120 | . | . | . | C | . | . | . | . | . | . | . | . | . | . | . | . | . | . | . | . | . | . | . |
| QH121 | . | . | . | . | . | . | . | . | . | . | . | . | . | . | . | . | . | . | . | . | . | . | . |
