# Supplementary material for: Cattle as Biological Indicators of Echinococcus granulosus Sensu Stricto in an Endemic Region from Chile
Source: Animals (Basel). 2026 Jun 19;16(12):1901. doi: 10.3390/ani16121901 (PMC13296003; doi:10.3390/ani16121901)
Supplement: Supplementary file 1 [file animals-16-01901-s001.zip › Supplementary File S2.pdf]

Supplementary Table S2. Samples of hydatid cysts collected from various sources in the Araucanía Region. Details include the name of the sample, the animal species from which it was collected, the organ, fertility, viability, whether cox1 markers were amplified, and the genotype or haplotype**.**

**Sample identification**	**Host**	**Geographic locality**	**Organ**	**Fertility**	***cox1***	**Genotype / Haplotype**
QH 01	Sheep	Cunco	Liver	+	+	G1
QH 02	Sheep	Cunco	Lung	+	+	$G1^{C}$
QH 03	Sheep	Villarrica	Liver	-	-	-
QH 04	Sheep	Curacautín	Liver	+	+	$G1^{C}$
QH 05	Sheep	Curacautín	Liver	+	+	$G1^{C}$
QH 06	Cattle	Nueva Imperial	Liver	-	-	-
QH 07	Sheep	Lautaro	Liver	+	+	G1
QH 08	Sheep	Melipeuco	Liver	+	+	$G1^{B}$
QH 09	Cattle	Victoria	Lung	-	-	-
QH 10	Cattle	Traiguén	Liver	+	+	G1
QH 11	Sheep	Villarrica	Liver	+	+	G1
QH 12	Pig	Villarrica	Lung	-	+	G1
QH 13	Pig	Villarrica	Liver	-	+	G1
QH 14	Cattle	Cholchol	Liver	-	+	$G1^{B}$
QH 15	Cattle	Cholchol	Liver	-	+	G1
QH 16	Cattle	Temuco	Liver	-	+	G1
QH 17	Cattle	Nueva Tolten	Lung	-	+	G1
QH 18	Cattle	Nueva Tolten	Liver	-	+	$G1^{C}$
QH 19	Cattle	Nueva Toltén	Liver	-	+	$G1^{B}$
QH 20	Cattle	Nueva Imperial	Liver	-	+	G1
QH 21	Cattle	Nueva Imperial	Lung	-	+	G3
QH 22	Cattle	Nueva Imperial	Liver	-	+	G1
QH 23	Cattle	Nueva Imperial	Lung	-	+	G1
QH 24	Cattle	Nueva Imperial	Hígado	-	+	G1
QH 25	Cattle	Nueva Imperial	Pulmón	-	+	G3
QH 26	Cattle	Freire	Liver	-	+	$G1^{B}$
QH 27	Cattle	Freire	Liver	-	+	$G1^{B}$
QH 28	Cattle	Nueva Imperial	Liver	-	+	G1
QH 29	Cattle	Lautaro	Lung	-	+	G1

QH 30	Cattle	Lautaro	Liver	-	-	-
QH 31	Cattle	Lautaro	Lung	-	+	G1
QH 32	Cattle	Lautaro	Liver	-	+	G1
QH 33	Cattle	Lautaro	Liver	-	+	G1
QH 34	Cattle	Cholchol	Lung	-	+	G1
QH 35	Cattle	Cunco	Liver	-	+	G1
QH 36	Cattle	Cunco	Liver	-	+	G1
QH 37	Cattle	Angol	Lung	-	+	G1[B]
QH 38	Cattle	Angol	Lung	-	+	G1
QH 39	Cattle	Angol	Lung	-	+	G1
QH 40	Cattle	Nueva Tolten	Lung	-	+	G1[C]
QH 41	Cattle	Nueva Tolten	Lung	-	+	G1
QH 42	Cattle	Nueva Tolten	Liver	-	+	G1
QH 43	Cattle	Nueva Tolten	Liver	-	+	G1[C]
QH 44	Cattle	Freire	Lung	-	+	G1
QH 45	Cattle	Freire	Liver	-	-	-
QH 46	Cattle	Pitrufquén	Lung	-	-	-
QH 47	Cattle	Pitrufquén	Lung	-	+	G1
QH 48	Cattle	Pitrufquén	Lung	-	+	G1
QH 49	Cattle	Curacautin	Liver	-	+	G1[B]
QH 50	Cattle	Nueva Imperial	Liver	-	+	G1[B]
QH 51	Cattle	Nueva Imperial	Liver	-	+	G1
QH 52	Cattle	Nueva Imperial	Lung	-	+	G1
QH 53	Sheep	Villarrica	Lung	+	+	G1
QH 54	Cattle	Cunco	Lung	-	+	G1
QH 55	Cattle	Nueva Imperial	Liver	-	+	G1
QH 56	Cattle	Nueva Imperial	Lung	-	+	G1
QH 57	Cattle	Nueva Imperial	Lung	-	+	G1
QH 58	Cattle	Nueva Imperial	Lung	-	+	G1
QH 59	Cattle	Nueva Imperial	Lung	-	+	G1
QH 60	Cattle	Nueva Imperial	Lung	-	-	-
QH 61	Cattle	Nueva Imperial	Liver	-	+	G1
QH 62	Cattle	Nueva Imperial	Lung	-	-	-
QH 63	Cattle	Galvarino	Lung	-	-	-
QH 64	Cattle	Nueva Imperial	Lung	-	+	G1[B]
QH 65	Cattle	Curacautin	Lung	-	+	G1[B]

QH 66	Cattle	Nueva Tolten	Liver	-	+	G1[B]
QH 67	Cattle	Freire	Liver	+	+	G1
QH 68	Cattle	Carahue	Lung	-	+	G1
QH 69	Cattle	Curacautin	Lung	-	+	G1[B]
QH 70	Cattle	Curacautin	Lung	-	+	G1[B]
QH 71	Cattle	Curacautin	Lung	-	+	G1[B]
QH 72	Cattle	Carahue	Lung	-	-	-
QH 73	Cattle	Villarrica	Liver	+	+	G1
QH 74	Cattle	Freire	Lung	-	+	G1
QH 75	Cattle	Freire	Lung	-	+	G1
QH 76	Cattle	Freire	Lung	-	+	G1
QH 77	Cattle	Vilcun	Lung	-	+	G1
QH 78	Cattle	Vilcun	Lung	-	+	G1
QH 79	Cattle	Vilcun	Lung	-	+	G1
QH 80	Cattle	Vilcun	Liver	-	+	G1
QH 81	Cattle	Vilcun	Lung	-	+	G1
QH 82	Cattle	PadreLas Casas	Liver	-	+	G1
QH 83	Cattle	Villarrica	Liver	-	+	G1
QH 84	Cattle	Curacautin	Liver	-	+	G1
QH 85	Cattle	Nueva Tolten	Liver	-	-	-
QH 86	Cattle	Nueva Tolten	Liver	-	+	G1[B]
QH 87	Cattle	Nueva Tolten	Lung	-	+	G1
QH 88	Cattle	Nueva Tolten	Lung	-	+	G1[B]
QH 89	Cattle	Nueva Tolten	Lung	-	+	G1[B]
QH 90	Cattle	Cunco	Liver	-	+	G1
QH 91	Cattle	Cunco	Lung	-	+	G1
QH92	Cattle	Angol	Lung	-	+	G1
QH 93	Cattle	Angol	Lung	-	+	G1
QH 94	Cattle	P Las Casas	Lung	-	+	G1[B]
QH 95	Cattle	Vilcun	Lung	-	+	G1
QH 96	Cattle	Vilcun	Liver	-	+	G1
QH 97	Cattle	Vilcun	Liver	-	+	G1
QH 98	Cattle	Villarrica	Lung	-	+	G1[B]
QH 99	Cattle	Villarrica	Lung	-	+	G1
QH 100	Cattle	Vilcún	Lung	-	+	G1
QH 101	Cattle	Loncoche	Liver	-	+	G1

QH 102	Cattle	Loncoche	Lung	-	+	G1
QH 103	Cattle	Loncoche	Lung	-	+	G1
QH 104	Cattle	Loncoche	Lung	-	+	G1
QH 105	Cattle	Cunco	Lung	-	+	G1
QH 106	Goat	Lonquimay	Lung	-	+	G1
QH 107	Sheep	Curacautín	Lung	+	+	G1
QH 108	Sheep	Curacautín	Liver	+	+	$G1^{B}$
QH 109	Sheep	Curacautín	Liver	-	+	G1
QH 110	Sheep	Lonquimay	Liver	+	+	G1
QH 111	Sheep	Lonquimay	Lung	+	+	G1
QH 112	Pig	Freire	Lung	-	+	G1
QH 113	Sheep	Loncoche	Liver	+	+	$G1^{B}$
QH 114	Sheep	Loncoche	Liver	+	+	G1
QH 115	Sheep	Gorbea	Liver	+	+	G1
QH 116	Sheep	PadreLasCasas	Liver	+	+	G1
QH 117	Pig	Loncoche	Liver	-	+	$G1^{B}$
QH 118	Goat	Curarrehue	Lung	-	+	G1
QH 119	Sheep	Vilcún	Liver	-	+	G1
QH 120	Sheep	Vilcún	Liver	-	+	$G1^{B}$
QH 121	Sheep	Vilcún	Liver	+	+	G1
